# Posterior fossa transient ischemic attack in the setting of bilateral persistent hypoglossal arteries: A case report and literature review

**DOI:** 10.1097/MD.0000000000027875

**Published:** 2021-11-12

**Authors:** Xiaolu Ren

**Affiliations:** a Department of Neurosurgery and Laboratory of Neurosurgery, Lanzhou University Second Hospital, Lanzhou, People's Republic of China; b Institute of Neurology, Lanzhou University, Lanzhou, People's Republic of China.

**Keywords:** internal carotid artery stenosis, persistent carotid-basilar anastomosis, persistent hypoglossal artery, transient ischemic attack

## Abstract

**Rationale:**

Persistent hypoglossal artery (PHA) is the second rare abnormal anastomosis of the internal carotid and vertebrobasilar arteries, and bilateral persistent hypoglossal arteries in particular have rarely been reported. This is the first case of bilateral persistent hypoglossal arteries presenting with posterior fossa transient ischemic attack (TIA).

**Patient concerns:**

We reported a 54-year old female with posterior fossa TIA due to the coexisting bilateral persistent hypoglossal arteries and left internal carotid artery stenosis.

**Diagnosis:**

The patient was diagnosed with posterior fossa TIA, bilateral persistent hypoglossal arteries and left internal carotid artery stenosis.

**Interventions:**

The patient was given aspirin 100 mg/qd and advised to avoid excessive neck movement.

**Outcomes:**

Symptoms of intermittent subjective dizziness accompanied by nausea were relieved.

**Lessons:**

Although requires no special treatment, PHA could be accompanied by hypoplasia of vertebral arteries and posterior communicating arteries and becomes the main blood supply pathway for the posterior circulation. Accurate identification and evaluation of PHA is important of ensuring the safety of carotid interventions and identifying specific types of stroke.

## 1 Introduction

About 20% to 25% of ischemic stroke occurs in the posterior circulation area. Posterior fossa transient ischemic attack (TIA) is a mild manifestation and symptom of posterior circulation ischemic stroke.^[1]^ It usually shows mild to moderate dizziness (47%), unilateral limb weakness (41%), gait ataxia (31%), dysarthria (31%), headache (28%), nausea or vomiting (27%), and blurred vision (20%), etc the above symptoms and signs can appear at the same time. Generally, it can recover completely within 24 hours without obvious sequelae.^[2]^ If it occurs repeatedly in a short time, this may indicate a higher risk of ischemic stroke.^[3]^ Atherosclerotic stenosis, dissection, dysplasia of vertebrobasilar system, dysplasia of circle of Willis and cardiogenic embolism are considered to be closely related to posterior fossa TIA.^[4]^ Persistent hypoglossal artery (PHA) is the second rare abnormal anastomosis between internal carotid artery (ICA) and vertebral artery (VA).^[5]^ PHA passes through the hypoglossal canal, connects the ICA and VA, and becomes the main pathway for blood supply to the posterior fossa. It is often accompanied by hypoplasia of VA and circle of Willis, so it is closely related to posterior circulation stroke.^[6]^ Only 7 cases of bilateral PHAs have been reported.^[7–13]^ We presented a case of bilateral PHAs which is characterized by posterior fossa TIA with dizziness as the main symptom. Except for moderate stenosis of left ICA, there are no other risk factors. The symptoms of this patient are considered closely related to bilateral PHAs.

## 2 Case report

**Figure 1:**
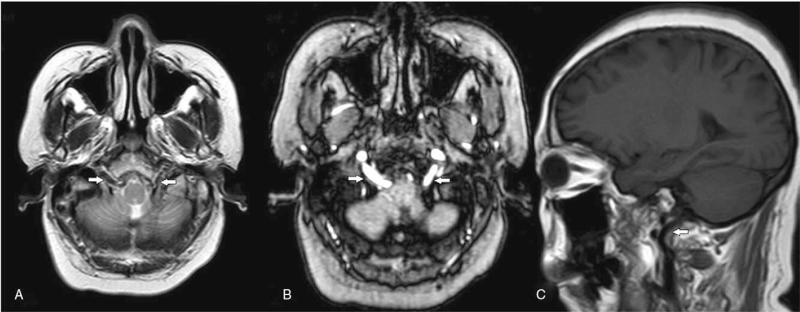
Brain MRI showing bilateral dilated hypoglossal canals, with PHAs (white arrows) traversing these (A, B). Right PHA (white arrow) originating from ICA and entering the skull through hypoglossal canal (C). MRI = magnetic resonance imaging.

**Figure 2:**
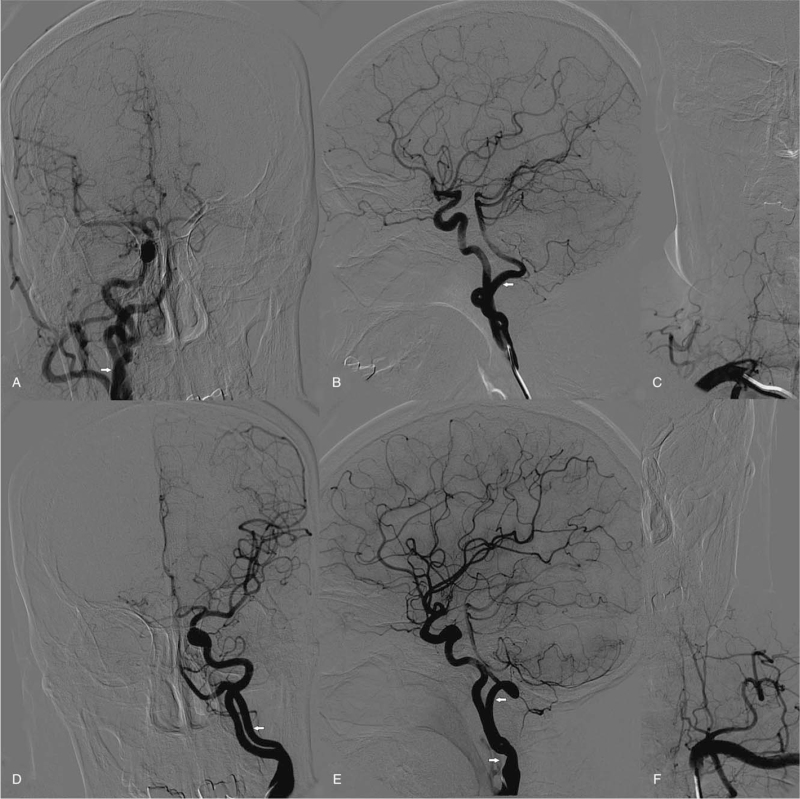
DSA of the right carotid artery (A, B), right subclavian artery (C). Left carotid artery (D, E) and left subclavian artery (F). Bilateral PHAs (white arrows) converging into intracranial segment of bilateral VAs before PICA takeoff, absence of extracranial segment of bilateral VAs, dysplasia of bilateral PCoAs, and moderate stenosis of 50% (E white arrow) of the proximal left ICA is shown. PCoAs = posterior communicating arteries, PICA = posterior inferior cerebellar artery.

A 54-year-old female was admitted with intermittent subjective dizziness accompanied by nausea for 2 days, which was aggravated upon neck flexion. She had no significant past medical history and no obvious neurological signs during physical examination. Cervical duplex ultrasound and transcranial Doppler suggested bilateral extracranial occlusion of the VAs, and compensatory blood supply from ICA to the vertebrobasilar system via distal collaterals. Magnetic resonance imaging showed normal intracranial segments of the bilateral VAs and basilar artery (BA). Also, bilateral expanded hypoglossal canals could be seen, particularly on the right side, through which arteries passed and communicated with the intracranial segment of the VA (Fig. 1). Digital subtraction angiography was performed to confirm bilateral PHAs supplying the intracranial segments of bilateral VAs before the posterior inferior cerebellar artery origins, absence of extracranial segment of bilateral VAs, dysplasia of bilateral posterior communicating arteries, and moderate stenosis up to 50% of the proximal left ICA (Fig. 2). Her symptoms were surmised to have been caused by TIA of the posterior circulation. The existence of bilateral PHAs and the related dysplasia of VAs and circle of Willis are considered to be the main cause.^[6,14]^ Left ICA stenosis may also be a cause because it may reduce the blood perfusion of ipsilateral VA or be accompanied by the shedding of microemboli.^[15]^ During hospitalization, 100 mg/d aspirin was given orally, and avoiding neck hyperactivity was recommended. The symptoms of dizziness and nausea gradually improved. The attack frequency decreased significantly and disappeared completely after 5 days. After evaluation, the risk of secondary severe ischemic stroke was considered to below and there was no indication of surgical treatment. The patient was discharge from hospital and was recommended take 100 mg/d aspirin orally after discharge and avoid excessive neck movement. After 6 months of follow-up, the patient complained of only occasional mild dizziness after discharge, which did not affect her normal life. There was no significant change in magnetic resonance imaging. She was recommended to maintain previous treatment scheme and keep long-term follow-up.

## 3 Literature review

In 1889, Batujeff first described the characteristics of PHA through autopsy. In 1954, Padget^[16]^ elucidated its embryonic development. Begg^[17]^ first confirmed the existence of PHA by cerebral angiography in 1961. Current literature reports that the incidence of PHA is about 0.027% to 0.260%.^[18]^ PHA is slightly more common in women and unilaterally on the left, while bilateral PHAs are extremely rare, with an incidence rate of 1.4% of PHA cases reported in the literature.^[19]^ We searched PubMed database, EMBASE, and Web of Science to review all bilateral PHAs reported in English literatures so far, using the following keywords as the search strategy: “bilateral”, “persistent hypoglossal artery”, “persistent carotid-basilar anastomosis”. Only 7 cases previously published were identified. The following data were extracted from relevant reports: author, publication year, age (year), sex, dominant PHA, vertebral arteries, imaging modalities, complication. Data were summarized in Table 1.^[7–13]^ Because the number of cases is too small, it is impossible to meta-analyze the data.

**Table 1:** Characteristics of bilateral PHAs reported.

Author	Publication year	Age (y)	Sex	Dominant PHA	Vertebral arteries	Imaging modalities	Complication
Karasawa et al^[7]^	1976	39	Male	Left	Left absent/right hypoplastic	DSA	SAH
Murayama et al^[8]^	1985	59	Male	Left	Hypoplastic	DSA	Aneurysm/SAH
Takahashi et al^[9]^	2012	76	Female	Left	Absent	MRA	None
Garge et al^[10]^	2016	60	Female	Left	Hypoplastic	DSA/MRA	Aneurysm/SAH
Patira et al^[11]^	2017	79	Male	Right	Absent	CTA/MRA	Thromboembolism
Choudhary et al^[12]^	2018	20	Female	None	Hypoplastic	CTA/MRA	None
Ozawa et al^[13]^	2019	87	Female	Left	Absent	MRA	None
Present case		54	Female	None	Absent	DSA/MRI	TIA

CTA = computed tomography angiography, DSA = digital subtraction angiography, MRI = magnetic resonance imaging, MRA = magnetic resonance angiography, SAH = subarachnoid hemorrhage.

## 4 Discussion

In the early stage of human development, when the embryo is about 4 to 5 mm in size, the primitive carotid artery supplies blood to the brain, paralleled with 2 longitudinal neural artery (LNA) on the dorsal side. LNA is not connected directly to the aorta but rather is supplied by the primitive carotid artery. There are 4 temporary anastomotic arteries between LNA and the primitive carotid artery. Excepting persistent proatlantal intersegmental artery (PIA), the other embryonic vessels are named according to the accompanying cranial nerves, including primitive trigeminal, otic and hypoglossal arteries. When the embryo is 5 to 6 mm in size, 2 LNAs gradually fuse to form the BA, and the PCoA gradually forms. Meanwhile, primitive trigeminal, otic and hypoglossal arteries gradually degenerate and disappear. PIA and PCoA continue to supply blood to the vertebrobasilar system from the carotid artery. When the embryo size is 7 to 12 mm in size, the VA gradually becomes fully developed, and the PIA regresses. If these anastomotic arteries do not regress in the embryo, a persistent internal carotid-basilar anastomosis will be formed.^[16,19]^ According to studies from autopsy and digital subtraction angiography, the incidence of persistent trigeminal artery is approximately 0.1% to 1.0%. PHA and PIA are lower still.^[5]^ Persistent otic artery is exceedingly rare, and previously only one angiographically-confirmed case has been documented.^[20]^

The distinction between PHA and type I persistent PIA is clinically important. Type I persistent PIA generally does not result in symptomatic nerve compression in the absence of aneurysms.^[19]^ Clinical symptoms associated with PHA compression include hypoglossal nerve (XII) paralysis and glossopharyngeal (IX) neuralgia.^[6,21,22]^ Several characteristics of PHA can help distinguish it from type I persistent PIA: PHA originates from the ICA at the level of C1 vertebrae or the intervertebral space of C1 to 2, which is higher than that of type I persistent PIA; PHA has a longer vertical ascending segment than type I persistent PIA; and PHA enters the skull through hypoglossal canal, while type I persistent PIA enters through the foramen magnum.^[23]^

PHA is believed to be directly associated with dysplasia of the VA and circle of Willis.^[19]^ If fetal VA dysplasia and/or poor fusion between the vertebral and basilar arteries occurs, a permanent carotid-basilar anastomosis and supply to posterior circulation may exist.^[24]^ In previous cases, if the primitive hypoglossal artery persists, ipsilateral VA and PCoA may be dysplastic, and contralateral VA and PCoA are present in only one third of cases.^[6,14,19]^ All these observations suggest that dysplasia of the VA may lead to persistence of primitive hypoglossal artery, and conversely PHA may induce dysplasia of the VA and PCoA. Therefore, the posterior part of circle of Willis is unstable in these patients, making PHA the most important blood supply route for the posterior circulation, and the ICA system the main blood supply source of the distal basilar, superior cerebellar, and posterior cerebral arteries. This implies that temporary blockage of a PHA during carotid surgery for instance, is associated with a higher risk of posterior circulation ischemia, and surgical or spontaneous internal carotid occlusion can lead to severe brainstem and cerebellar infarction.^[6,14]^

The junction of PHA with the ICA may have similar anatomic and hemodynamic structure with a normal carotid artery bifurcation. Theoretically however, this region may have a higher risk of atherosclerotic plaque formation, leading to ICA stenosis. Although atherosclerotic plaques rarely affect the PHA proper, dislodged thrombi may cause embolization not only in anterior circulation but also in posterior circulation.^[15]^ In this patient, the coexisting bilateral PHAs and left ICA stenosis may explain the occurrence of posterior fossa TIA symptoms. Thus, discovery of a PHA is of great significance in explaining mechanisms of cerebral hypoperfusion and infarction in different territories from the same artery.^[25]^ The possibility of carotid-BA anastomosis should be considered in clinical cases of cerebral infarction in multiple territories not adequately explained by cardioembolism or other such mechanisms. In the presence of carotid-basilar anastomosis, if the ICA exhibits severe stenosis or occlusion, the BA may also reverse blood flow to compensate for the internal carotid system.^[26]^

Additionally, some investigators believe that the PHA may have defects in the development of its medial layer, thus exposing the trunk of BA to abnormal hemodynamic pressure and contributing to aneurysm formation.^[27,28]^ There is even evidence showing PHA is related to Moyamoya disease.^[29,30]^

## 5 Conclusion

PHA is a rare carotid-vertebrobasilar artery anastomosis, and bilateral PHAs are exceedingly rare. PHA is often accompanied by hypoplasia of VA and PCoA, and it may be the main blood supply pathway for the posterior circulation. Accurate identification and evaluation of PHA is important of ensuring the safety of carotid interventions and identifying specific types of stroke.

## Acknowledgments

The author thank the patient for consenting to the publication of this case.

## Author contributions

**Conceptualization:** Xiaolu Ren.

**Data curation:** Xiaolu Ren.

**Formal analysis:** Xiaolu Ren.

**Investigation:** Xiaolu Ren.

**Writing – original draft:** Xiaolu Ren.

**Writing – review & editing:** Xiaolu Ren.
